# Negative impact of single housing on chronic kidney disease mineral and bone disorder outcomes

**DOI:** 10.1093/jbmrpl/ziag160

**Published:** 2026-10-24

**Authors:** Erica L Clinkenbeard, Jo Wanner, Laila Mansour, Katharina M Kirschner, Sophia Kroll

**Affiliations:** Department of Medical and Molecular Genetics, School of Medicine, Indiana University, Indianapolis, IN 46202, United States; Indiana Center for Musculoskeletal Health, Indiana University, Indianapolis, IN 46202, United States; Department of Medical and Molecular Genetics, School of Medicine, Indiana University, Indianapolis, IN 46202, United States; Indiana Center for Musculoskeletal Health, Indiana University, Indianapolis, IN 46202, United States; Department of Sciences, John Jay College of Criminal Justice, The City University of New York, New York, NY 10019, United States; Department of Medical and Molecular Genetics, School of Medicine, Indiana University, Indianapolis, IN 46202, United States; Indiana Center for Musculoskeletal Health, Indiana University, Indianapolis, IN 46202, United States; Department of Medical and Molecular Genetics, School of Medicine, Indiana University, Indianapolis, IN 46202, United States

**Keywords:** chronic kidney disease, male, osteoblast, bone, C57BL/6

## Abstract

Chronic kidney disease (CKD) is a disorder of acquired disrupted mineral metabolism that affects millions in the United States and has no cure. Due to disturbed mineral homeostasis bones are particularly affected showing substantial bone loss during CKD and intricately linked with morbidity and mortality. Animal models serve as a crucial platform to investigate and elucidate the tissue cross-communication that facilitates pathogenic changes during CKD. One preclinical rodent model gaining in popularity is the adenine-diet induced CKD, which negates the requirement for highly technical surgical interventions and displays several of the CKD disrupted mineral metabolism hallmarks observed in patients. Interestingly, although CKD alone can negatively impact bone, other factors, such as individual housing, can impair normal bone homeostasis. This could be encountered experimentally especially when generating experimental animals in-house, in particular, using males that maintain aggressive territorial actions. We sought to evaluate the impact of reduced cage housing numbers, replicating potentially encountered cage number conditions, on the progression of CKD. We hypothesized that single housing alone would accelerate bone loss and therefore exacerbate CKD mineral dysregulation and related outcomes. To test this, C57BL/6J male mice at 10 wk old were maintained at 5-, 3-, or 1-mice per cage while receiving casein-control or the CKD-inducing adenine diet. We found that within 4 wk of diet administration, individually housed mice had more profound weight loss corresponding with increased circulating urea, hyperphosphatemia, and bone loss compared to mice maintained at 5 per cage. Additionally, 50% of the individually housed CKD animals developed cardiovascular calcifications, a sequelae of impaired mineral excretion. These data demonstrate housing density significantly impacts outcomes related to CKD and an important variable to consider when conducting basic and translational mouse CKD studies.

## Introduction

Chronic kidney disease (CKD) is a common acquired disorder affecting millions in the US and abroad.^1^ The kidney is the primary organ responsible for excretion of excess minerals, including calcium and phosphate. Impaired handling of these minerals during CKD causes pathogenic rises in calcium and phosphate regulating hormones PTH as well as fibroblast growth factor 23 (FGF23), substantial bone loss through altered turnover status, and ultimately increased indices for cardiovascular calcifications.^2^^,^^3^ Either changed individually or in combination are characteristics of mineral and bone disorder of CKD (CKD-MBD), for which there is no cure. Thus, there is a tremendous need to improve our basic understanding of the underlying mechanisms for disease progression to identify novel modalities for therapeutic intervention.

Understanding CKD has been facilitated with utilization of rodent models for basic and translational studies through a variety of genetic and acquired approaches.^4^ One methodology gaining in popularity is adenine diet (AD) feeding in mice. Previously, this approach was restricted to rats as mice have a natural taste aversion to adenine contained within food. In 2013, Jia et al. found that placement of 0.15%-0.3% adenine could be adequately disguised in casein-based pellets for mouse consumption.^5^ Resulting adenine ingestion induces crystal accumulation in the tubule, causing damage with subsequent inflammation and replacement of nephrons with fibrotic tissue, also known as tubulointerstitial fibrosis. This correlates to serum accumulation of typically excreted products, such as creatinine and urea: hallmarks of reduced kidney filtration. Importantly, the model also displays features of CKD-MBD with elevations in PTH and FGF23, as well as high bone turnover with robust osteoclast activation leading to bone loss especially observed with cortical porosity.^5^^,^^6^ These parallel observations in patients with CKD, thus rendering this method an attractive model for basic and translational research.

In contrast to technical demands of surgical approaches, the AD model generates consistent CKD markers within a relatively rapid timeline between 4 and 8 wk depending on the concentration of adenine content and other experimental factors.^7^ One of those factors includes sex, as male mice and rats are known to have a more rapid progression of CKD compared to females.^8^^,^^9^ It is important to consider that when running experiments with genetically modified animals, researchers generate experimental mice in-house, and once male mice are separated, detrimental fighting will occur if attempting to re-house males together.^10^^,^^11^ Thus, it is possible that some CKD experimental animals are individually housed. Recent reports suggest individual housing is deleterious for bone, an important component of mineral homeostasis.^12^ Thus, we hypothesized that decreased cage density accentuates bone loss and thereby exacerbates disrupted mineral handling thereby contributing to CKD-MBD. We empirically tested the effects of maintaining WT C57BL/6 male mice housed at 5-, 3-, and 1-per cage on early CKD parameters through 4 wk of diet administration.

## Materials and methods

### In vivo CKD induction

All animal studies were performed in accordance with approved Indiana University School of Medicine IACUC protocol (Protocol #25043). Lights within the Laboratory Animal Research Center (LARC) were maintained in a standard 12-h cycle, and animals had constant access to food and water. C57BL/6J male mice were purchased (JAX #000664) at 9 wk of age and allowed to acclimate for 1 wk. Purchased animals were housed together at 5 mice per cage during this acclimation period. At 10 wk, mice were weighed and stratified for equivalent body weight across groups. For individual housing, mice were removed from the original cage containing 5 mice thereby reducing it to three mice per cage. The two removed mice were then each housed individually. All mice maintained as individually housed received extra bedding and a housing hut to reduce stress as part of the LARC requirements and IACUC protocol. Cohorts of mice were placed on either casein-based control diet (0.9% phosphorus and 0.6% calcium; Inotiv) or casein base pellets with 0.2% adenine (0.9% phosphorus and 0.6% calcium; Inotiv) to induce CKD.^5^ We collected material from 3 separate cohorts of mice which included casein- and adenine-fed cages to limit batch effects. We also maintained animals at all 3 density levels to ensure at least 2 batches of animals were compiled for the different cage density groups. Mice were weighed weekly for a total of 4 wk of diet administration. To monitor food intake for the 5- and 1/cg groups, food pellet weights were collected every 3-4 d. These weights were then normalized to number of days between weight measurements and number of mice in the cages. Prior to necropsy, mouse body condition score (1-5) was evaluated based on similar parameters for rats whereby 3/5 indicates appropriate conditioning.^13^ Additionally, calcein-AM (30 mg/kg; Sigma-Aldrich) and Alizarin Red-S (20 mg/kg; Sigma-Aldrich) fluorochromes were intraperitoneally injected 6- and 1-d prior to necropsy, respectively to evaluate bone formation rates.

### Serum biochemistries

At necropsy, the blood was collected via right ventricular cardiac puncture and processed via centrifugation for serum, with a portion being initially collected in plasma EDTA coated tubes (R&D). IU Translational Core (Roche Diagnostics) analyzed the isolated serum for calcium, phosphate, alkaline phosphatase, total iron, and blood urea nitrogen (BUN). The technician in the core facility was blinded to the group designation for the animal number provided. Intact FGF23 and intact PTH(1-84) concentrations were measured using ELISAs (Quidel) from mouse plasma samples according to the manufacturer’s instructions. Cystatin C ELISA was used as another parameter for evaluating renal filtration (Abcam). Absorbance at 450 and 620 nm wavelengths detected on Molecular Devices SpectraMax i3 spectrophotometer collected the absorbance values for standards and samples. Sample concentrations were calculated based on ELISA standard curves.

### Micro-CT

Femora were cleaned of soft tissue, fixed in 4% PFA, and transferred to long term storage in 70% ethanol after 2 d. Bones were scanned and processed similar to previous publications.^14^^,^^15^ Briefly, a 3D printed holder to image groups of three bones within one scan using an isotropic voxel size of 8 μM in a Skyscan 1272 (Bruker).^16^ Scans were performed through a 0.5 mm aluminum filter with a 0.7° angle step and 2 frames averaged. Prior to scans, a phantom calibration was conducted using hydroxyapatite standards (0.3 and 1.25 g/cm^3^ CaHA). nRecon software was used for scan reconstructions, and DataViewer was used to rotate each bone to a consistent orientation for analysis. Trabecular ROIs were selected at 1 mm below the growth plate and cortical bone ROIs were selected at the midpoint for the entire length of the femur. CT Analyzer software (CTan) quantified standard cancellous bone architecture parameters of trabecular thickness, spacing, number, and bone volume over tissue volume. Cortical bone was processed with 2-dimensional (2D) analysis for thickness and area while 3D analysis determined % porosity. Analysis occurred in a blinded fashion until all of the raw data was collected.

### RNA analysis

After necropsy, bone tissue was immediately processed after necropsy, whereas kidney tissue was stored at −20 °C in RNALater (Thermo Scientific). For extraction, tissues were placed in NAVY beaded tubes (Midwest Scientific) along with Trizol (Thermo Fisher) followed by homogenization in the Bullet Blender (Midwest Scientific) for 2 rounds of 5 min at maximum rotation. The remainder of the Trizol extraction proceeded according to the manufacturer’s instructions. Resuspended RNA, regardless of tissue source, underwent column cleanup using the BioLine RNA extraction kit according to the manufacturer’s instructions. All RNA was quantified using Nanodrop 2000 (Thermo Scientific). 50 ng RNA was analyzed with the TaqMan OneStep RNA to Ct kit (Life Technologies) using TaqMan primer/probe mix (Life Technologies) for the following genes: *Bglap*, *Alpl*, *Fgf23*, *Dmp1*, *Il-6*, and *Col1a1*, which were run on a Quant Studio 3 Real-Time PCR instrument (Life Technologies). Mouse tissue mRNA targets of interest were normalized with hypoxanthine guanine phosphoribosyl transferase (*Hprt*) using the 2^−Δ/ΔCt^ calculation method. A list of primer/probe information is available upon request. This procedure occurred in a blinded fashion until all of the raw data was collected.

### Histology

All tissues were embedded, sectioned, and stained within the IU Histology Core. Femurs, fixed in 4% paraformaldehyde, after micro-CT scanning were embedded without decalcification in MMA. 5 μM longitudinal sections of the distal femur were stained for von Kossa using Fast Green counterstain. Femur midshafts were also MMA-embedded for cross-section collections. For dynamic histomorphometry Fluorescent analysis of calcein-AM (green) and alizarin red-S (red) incorporation within the bone was determined at the periosteal and endocortical surfaces using OsteoMeasure high resolution digital video system (OsteoMetrics, Inc.) similar to.^17^ Abbreviations and units are consistent with established guidelines.^18^ Whole hearts and right kidneys fixed in 4% PFA for 24 h were paraffin embedded. 5 μM coronal sections were also stained with von Kossa similar to the distal femurs to capture aberrant calcifications. Serial sections for heart and kidney were also stained with Masson’s Trichrome for visualizing fibrosis. Kidney tissue sections were also stained with H&E for visualizing general morphology and to identify any potential cell infiltrates. All histology images were captured using the Keyence BZ-X810 microscope with collection of 4× objective images. Multiple images were captured to collect the entirety of the histological section and subsequently stitched together to form a singular compiled TIFF file using the Keyence BZ-H4A/Advanced Analysis Software. Kidney von Kossa and Masson’s Trichome stained images were processed using ImageJ (Fiji) to determine percentage of calcified tissue and fibrosis within the tissue, respectively. To accomplish this, color threshold was used to define the total area of the tissue. From here, with von Kossa images, the threshold was adjusted to exclusively define the black positive von Kossa staining within the section. Area of von Kossa positive stain was normalized to total area. For Masson’s Trichrome, the color hue was adjusted to 148-187 to isolate the area specific for collagen. The area of positive blue staining was normalized to the total area.

### Statistical analysis

Data are presented as individual biological replicate data points along with mean + SE. One way ANOVA was used to compare more than 3 independent groups. Two-way ANOVA was used when Gaussian distribution was normal for statistical analysis between densities/diet. If significant values were obtained, Tukey’s post-hoc multiple comparisons were performed. Threshold cutoff for a significant *p*-value was designated at *p* < .05 (GraphPad Prism). Tables indicating significant effects of each independent variables (diet or cage density) as well as interaction (diet × cage) based on the initial 2-way ANOVA are provided below the graphs.

## Results

Immediately prior to segregating mice to cages with 5-, 3-, or 1- per cage (5/cg, 3/cg, and 1/cg), body weights were collected. To determine whether the cage density had an impact on overall body condition of the mice, we monitored body weight on a weekly basis. As previously seen and expected, all mice receiving AD to induce CKD exhibited weight loss from baseline compared to casein control (Cas). Interestingly, beginning at 1 wk of diet administration, a cage density separation became apparent whereby AD-fed 1/cg mice had significantly lower body weight compared to the AD-fed 5/cg mice. After 4 wk of diet administration, mice AD-fed mice had significantly lower weight compared to both baseline as well as Cas-fed mice at the same cage density. The AD-fed cage density showed further separation; however, the body weight 1/cg group was significantly lower to only the AD-fed 5/cg mice (Figure 1A). Importantly, one Cas-fed group had moderately lower body weight at initiation of the study and thus, we also calculated the % body weight changes throughout the study compared to baseline. As expected, Cas-fed mice retained positive body weight changes over time and were not significantly different between cage densities. Additionally, AD-fed showed a negative body weight % change over time. Beginning after 1 wk of diet administration the AD-fed 1/cg group had more profound body weight change vs AD-fed 5/cg, which was sustained throughout the study timeline (Figure 1B). We measured food intake to determine if cage density affected this activity especially between the 5/cg and 1/cg densities. Throughout the study we found consistently, mice provided with adenine exhibited lower food consumption compared to casein-fed groups when comparing between similar cage densities. Interestingly, at week 1 and 2 of diet administration, 1/cg mice consumed higher food amounts compared to the 5/cg group. By week 3, the Cas-fed mice ate more food than AD-fed cages, yet there were no differences between cage densities (Figure 1C). We also determined body score for the mice prior to necropsy based on parameters assessed with the IU Laboratory Animal Research Center. All Cas-fed mice exhibited a normal body score of 3/5 after 4 wk of diet administration (Figure 1D). Additionally, AD-fed 5/cg mice had minimal changes in body score that were not significantly different from Cas-fed mice. Adenine-diet-fed 3/cg and AD-fed 1/cg mice, however, showed a density specific reduction in body score, demonstrating a significant interaction between diet and cage density with 2-way ANOVA (*p* < .0001). Hunching, lethargy, and prolapsed penis were the most notable contributors to the body score for the AD-fed 1/cg mice. Thus, these data demonstrate differential phenotypic outcomes for AD-fed mice based on mouse number cage density.

**Figure 1:**
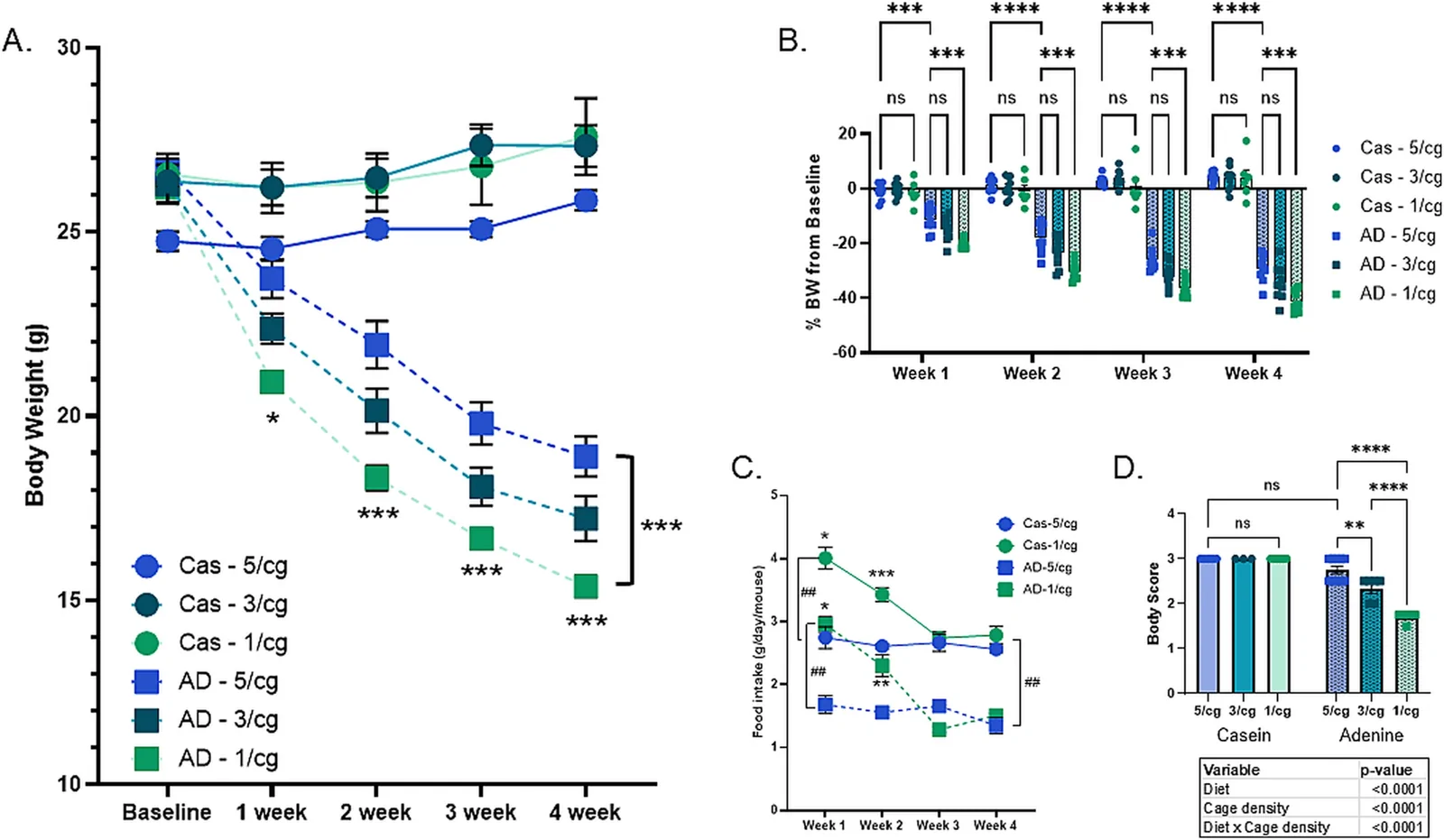
Cage density negatively impacts body weight and condition with CKD. (A) Body weight collected at the start of diet administration and throughout 4 wk of diet administration (^*^p < .05 and ^***^p < .001). (B) Calculated percent change of body weight at each time point of diet administration compared to baseline (^***^p < .001 and ^****^p < .0001). (C) Food intake measurements for 5/cg and 1/cg groups (^*^p < .05 and ^***^p < .001 1/cg vs 5/cg same diet; ^##^*p* < .01 Cas-fed vs AD-fed, same cage density). (D) Body condition score of each group at conclusion of the 4 wk diet study (^**^p < .01 and ^****^p < .0001) with 2-way ANOVA table outlining individual contributions of each variable (*n* = 7-10 per group).

To determine if differences in overall body weight and appearance translated to differences in kidney function and mineral homeostasis, we performed serum biochemistry analysis. Consistent with AD inducing CKD, BUN was significantly elevated in AD-fed mice compared to Cas-fed mice (*p* < .0001). There was no impact of cage density for any of the Cas-fed groups; however, there was a notable cage density dependent increase of serum BUN in AD-fed 1/cg mice compared to both AD-fed 3/cg and 5/cg, indicating a significant interaction with diet and cage density (*p* < .0003, Figure 2A). For minerals, calcium remained stable across the Cas-fed groups as well as the AD-fed 5/cg and 3/cg groups. In measuring serum Cystatin C levels, we found no differences between diets or cage densities (Figure 2B). Curiously, there was a significant reduction in serum calcium for the AD-fed 1/cg mice (Figure 2C). Serum phosphate showed a significant effect with diet (*p* < .0001) consistent with elevated phosphate with CKD. Examining cage number effects phosphate levels were not significantly changed in the AD-fed 5/cg vs the Cas-fed group, whereas there was a significant rise in phosphorus in the AD-fed 3/cg and −1/cg groups compared to the AD-fed 5/cg mice (Figure 2C). Although there were no significant differences between these 2 groups, the effect elicited a significant diet and cage density interaction (*p* = .0075, Figure 2D). Anemia is often a secondary outcome of CKD; thus, it is consistent that a significant diet effect was calculated (*p* < .0001) with reductions in serum iron detected in AD-fed 5/cg and 3/cg mice compared to Cas-fed groups (Figure 2E). However, serum iron levels in the AD-fed 1/cg group were indistinguishable from the Cas-fed 1/cg mice, suggesting difference of iron handling in these animals. Finally, we assessed serum alkaline phosphatase (Alk Phos) which can be used as a marker for osteoblast activity. A significant diet effect was observed (*p* = .0043) as well as a diet with cage density interaction (*p* = .0029), yet the only significant difference observed was an increase in the AD-fed 1/cg group compared to AD-fed 5/cg and Cas-fed groups (Figure 2F). These data demonstrate a parallel between body weight loss and serum biochemistry parameters that are consistent with worsened CKD outcomes.

**Figure 2:**
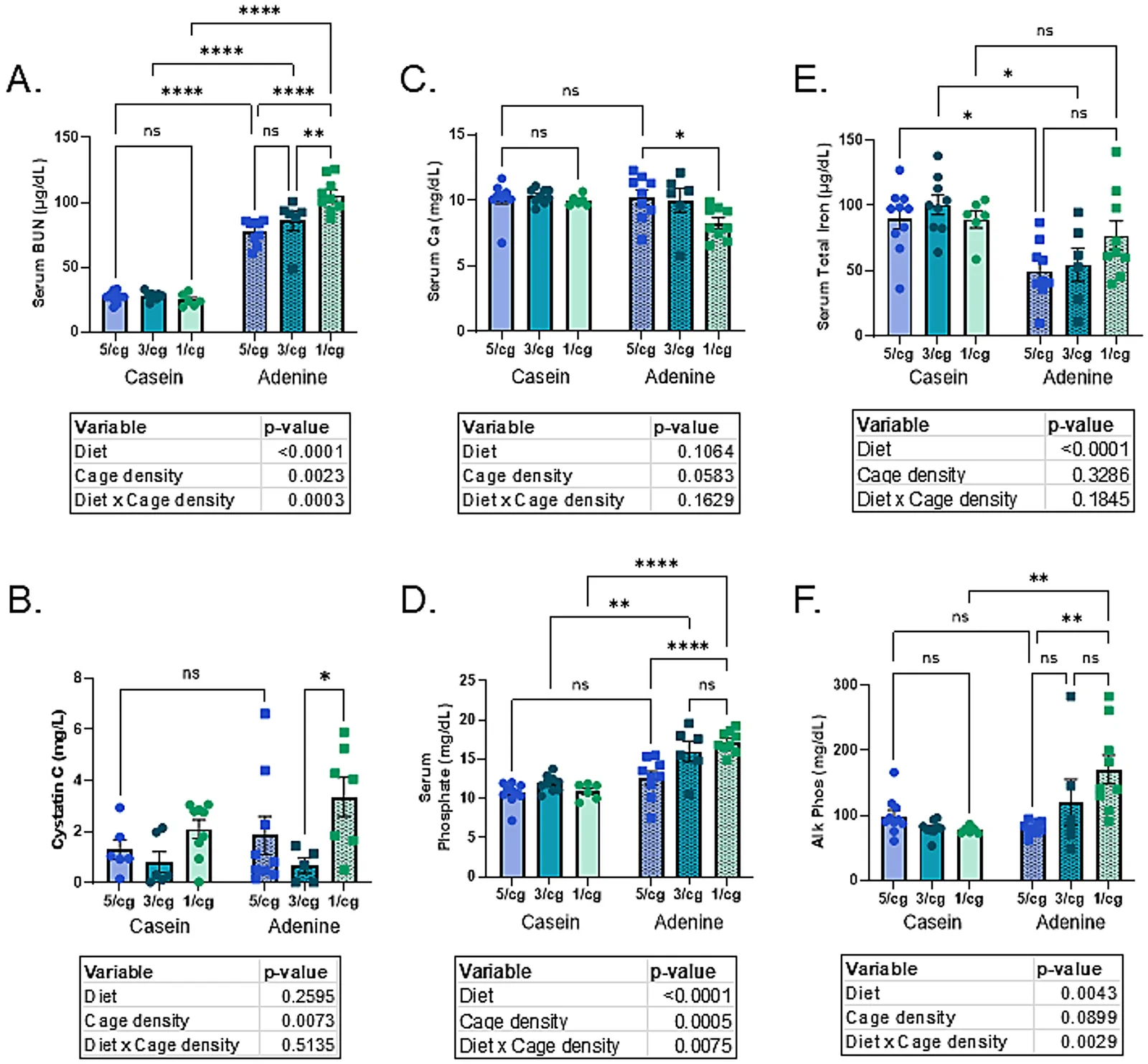
Cage density promotes CKD as observed with serum biochemistries. Serum analysis for (A) blood urea nitrogen (BUN), (B) cystatin C, (C) calcium, (D) phosphate, (E) total iron, and (F) alkaline phosphatase (Alk Phos) with corresponding 2-way ANOVA tables below (*n* = 5-9 per group; ^*^p < .05, ^**^p < .01, and ^****^p < .0001).

To rule out the possibility of enhanced fibrosis and inflammation within the kidney driving the serum BUN and phosphate levels, we evaluated expression levels of type 1 collagen (*Col1a1*) and inflammatory cytokine interleukin 6 (*Il-6*), respectively. As anticipated with CKD, both markers showed a significant diet effect (*p* < .0001), whereby AD-fed groups had elevated *Col1a1* and *Il-6* mRNA over Cas-fed groups. No cage density effects were noted, and therefore, no significant changes in expression level were observed between the different housing groups (Figure 3A and B). We next evaluated the kidney through histological evaluation. Serial sections stained with H&E, Masson’s Trichrome, and von Kossa were used to visualize morphology, fibrosis, and calcification, respectively (Figure 3C). As expected, Cas-fed 1/cg showed no fibrosis and was elevated with AD-fed mice. For this parameter, although there was a modest increase in fibrotic percentage area, there were no significant differences between cage numbers (Figure 3D). Similarly, no von Kossa calcification was evident in Cas-fed mice. For this parameter, there was a significant increase in positive calcified area % for kidneys from the AD-fed 1/cg group compared to the AD-fed 5/cg mice (Figure 3E).

**Figure 3:**
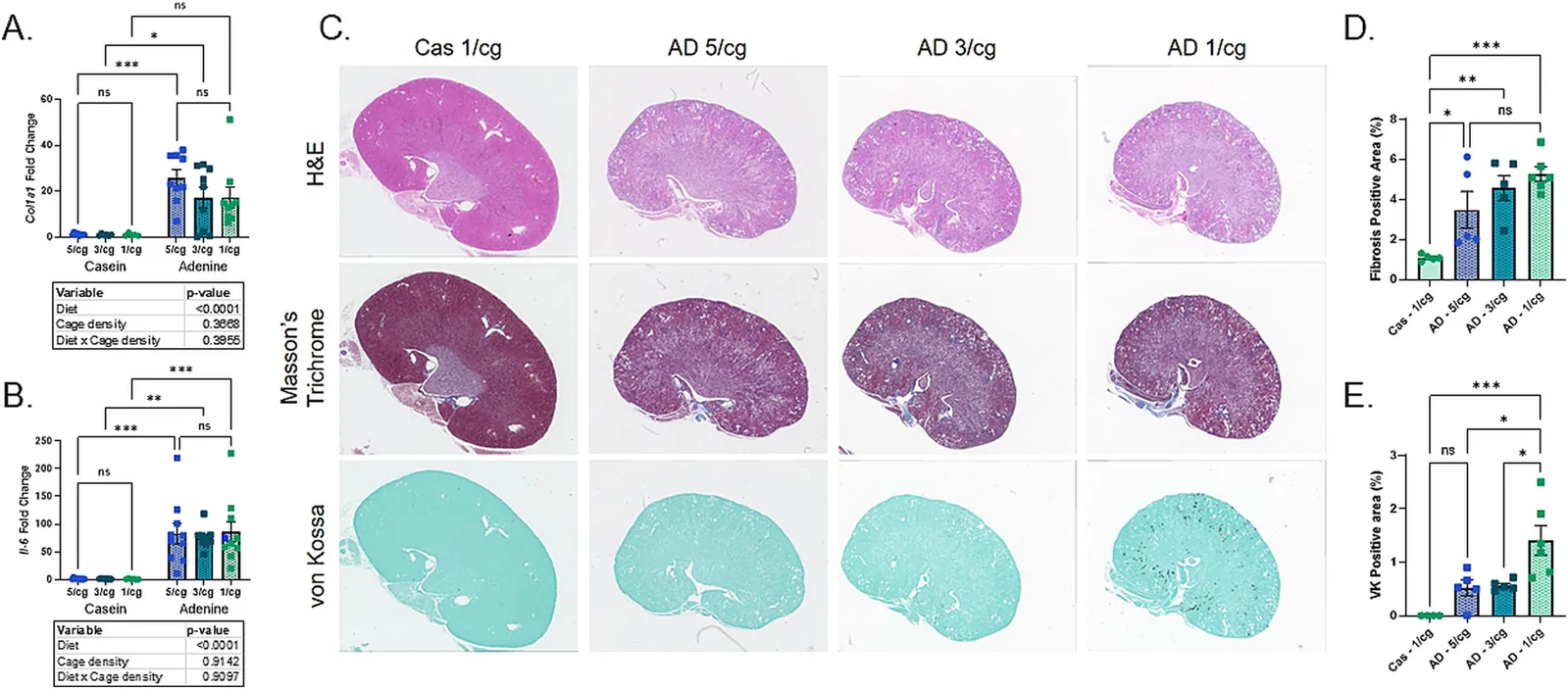
Cage density influences kidney morphology. RNA analysis of whole kidney expression for (A) *Col1a1* and (B) *Il-6* mRNA after 4 wk of diet administration (*n* = 6-9). (C) Representative images for kidney histology sections for H&E, Masson’s trichrome, and von Kossa. Quantification of (D) fibrosis positive area and (E) von Kossa positive area from the whole kidney tissue (*n* = 4-5; ^*^p < .05, ^**^p < .01, and ^***^p < .001).

Due to the significant differences in serum calcium and phosphorus, we measured the corresponding regulatory hormones within the circulation. Full length, intact FGF23 (iFGF23) was measured and found to correspond to the elevations in serum phosphorus in the AD-fed 5/cg and 3/cg groups compared to Cas-fed mice. Paradoxically, iFGF23 was substantially lower in the AD-fed 1/cg mice compared to AD-fed 3/cg mice and was not statistically different compared to Cas-fed groups (Figure 4A). PTH was measured as the primary calcium regulator and found to increase in the AD-fed mice consistent with secondary hyperparathyroidism during CKD. Interestingly, there were no observed significant differences driven by cage density within the AD-fed groups despite a significant cage density effect calculated in the 2-way ANOVA (Figure 4B).

**Figure 4:**
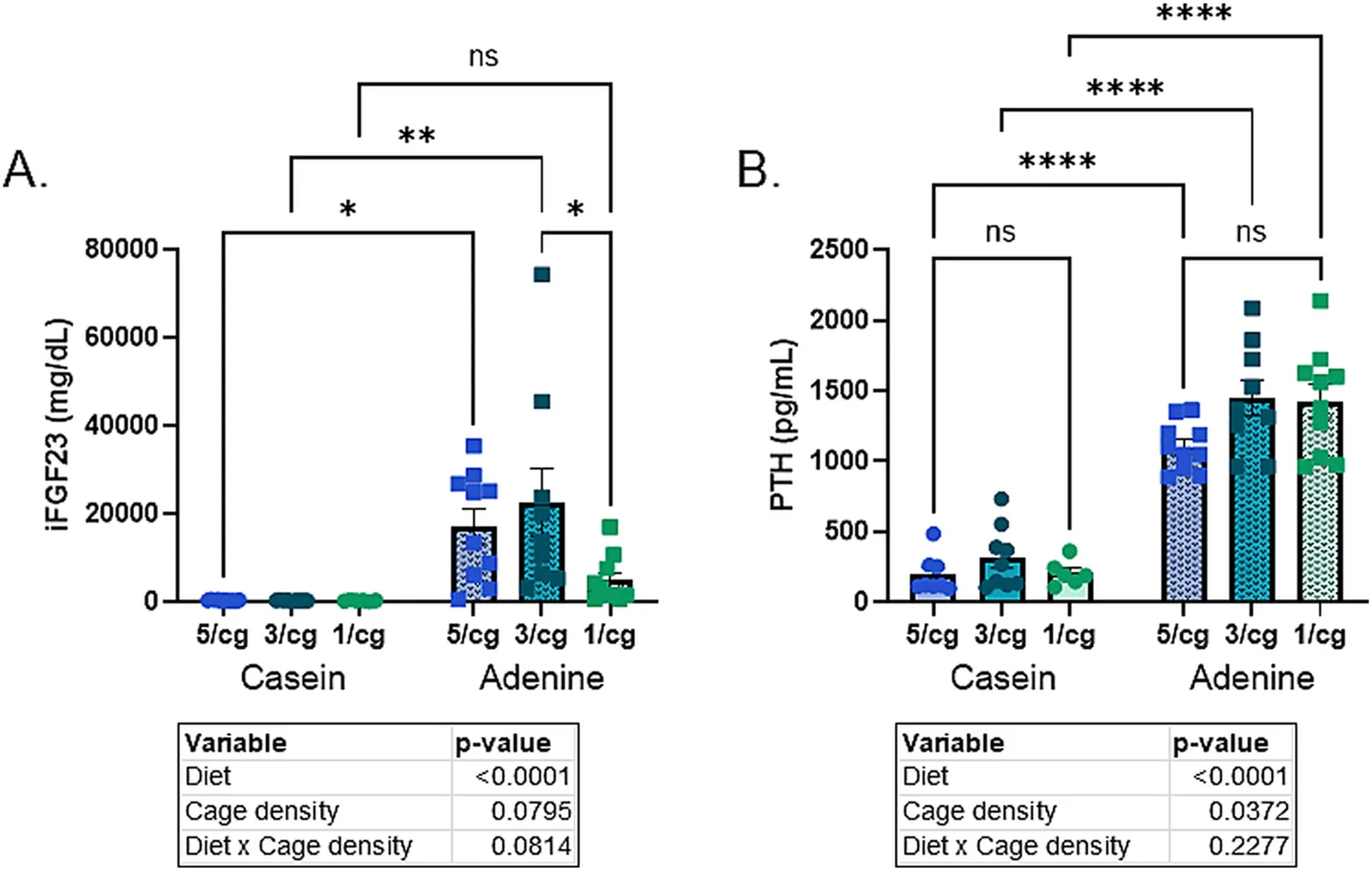
Changes in mineral metabolism hormones with CKD. Serum analysis for (A) intact FGF23 (iFGF23) and (B) PTH in casein and adenine-fed mice with corresponding 2-way ANOVA tables below (*n* = 6-10 mice per group; ^**^p < .01, ^***^p < .001, and ^****^p < .0001).

The primary outcome for the study was to evaluate the impact of cage density on CKD-MBD bone loss, and we first measured this through osteoblast RNA expression within the tibia. Osteocalcin (*Bglap*) mRNA showed a significant diet effect (*p* < .0001) with a more modest diet by cage density interaction (*p* = .0435). Interestingly, there was an unexpected *Bglap* 3-fold increase in the Cas-fed 1/cg group compared to Cas-fed 5/cg. In contrast, AD-fed mice demonstrated a reduction in *Bglap* mRNA such that AD-fed 1/cg levels were significantly lower than the Cas-fed 1/cg mice (Figure 5A). Alkaline phosphatase (*Alpl*) mRNA had no dietary or cage density effect despite an upward trend within the AD-fed mice (Figure 5B). As expected, *Fgf23* mRNA was significantly elevated 25- to 40-fold in the AD-fed mice compared to Cas-fed groups showing a significant diet effect (*p* < .001). However, in contrast to the iFGF23 data, there was no difference observed within the different cage density groups (Figure 5C). Dentin matrix protein 1 (*Dmp1*) mRNA, a late osteoblast marker, also showed no significant differences between either diet or cage density (Figure 5D). These data demonstrate that cage density had minimal effects on the osteoblast gene expression signature within the bone during CKD.

**Figure 5:**
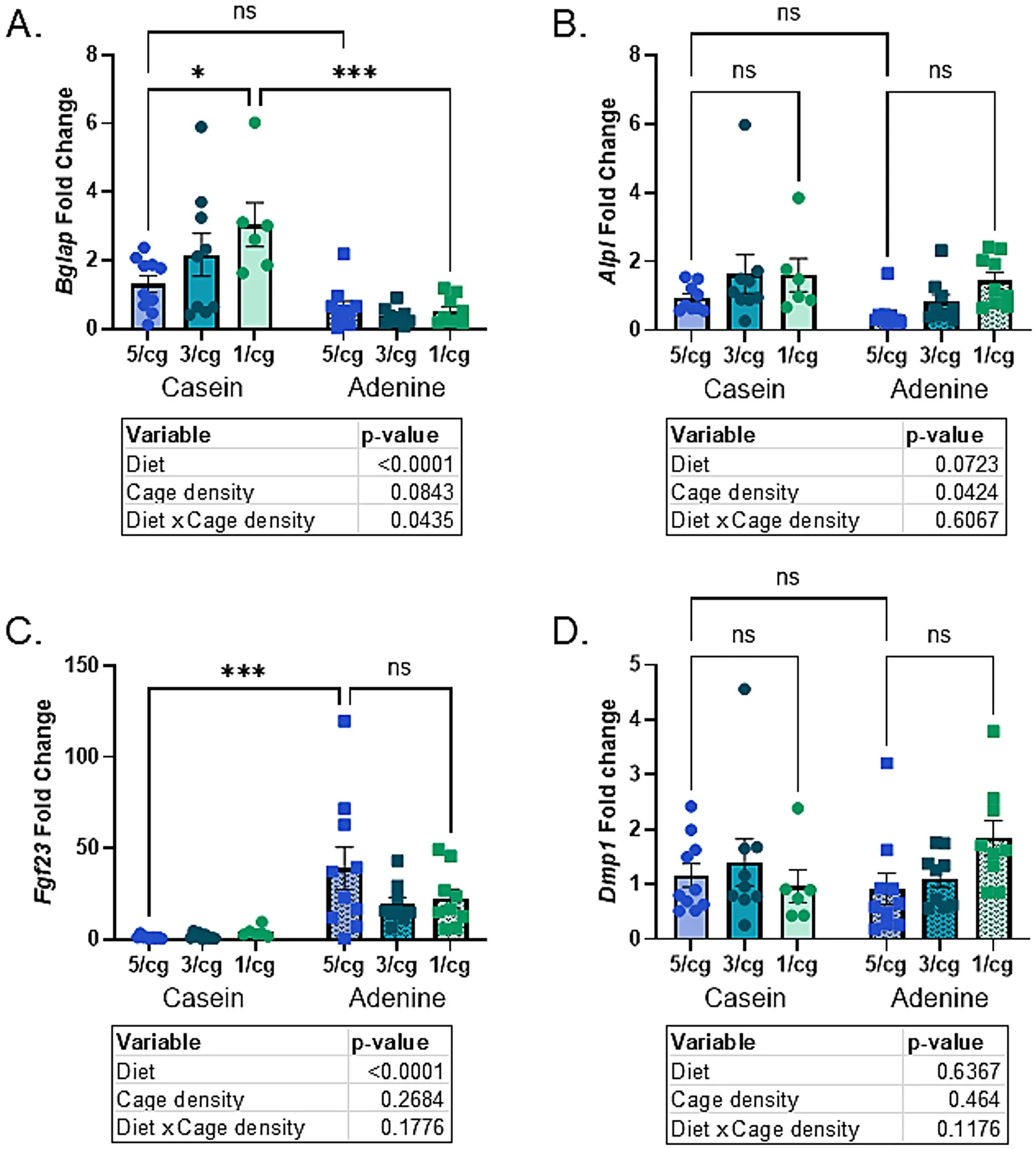
Cage density exerts minimal effects on bone RNA expression. Whole tibia RNA analysis for (A) *Bglap*, (B) *Alpl*, (C) *Fgf23*, and (D) *Dmp1* mRNA. Corresponding 2-way ANOVA tables are presented below (*n* = 5-9 per group, ^*^p < .05 and ^***^p < .001).

To determine if the mice exhibited phenotypic changes in bone during the 4-wk diet study, we evaluated distal femur micro-CT measurements for both trabecular and cortical bone compartments. Trabecular bone volume compared to tissue volume (BV/TV%) showed a significant diet effect with 2-way ANOVA (*p* = .0156). However, no significant individual differences between diets were found (Figure 6A). Trabecular thickness (Tb.Th) was determined to have a significant cage density effect (*p* = .0121). Despite a modestly lower Tb.Th level in the 1/cg groups, comparisons vs 5/cg were not significantly different (Figure 6B). Trabecular number (Tb.N) showed a significant diet effect (*p* < .0001), yet the only significant difference was observed between AD-fed 5/cg and Cas-fed 5/cg groups (Figure 6C). Interestingly, trabecular separation (Tb.Sp) failed to demonstrate any differences based on diet or density (Figure 6D). Representative von Kossa images of the distal femur align with the micro-CT data (Figure S1A). Although not quantified, there appeared to be a cage density affect in adipocytes within the adenine-fed distal femur marrow space. We next evaluated the cortical compartment and found a significant reduction in cortical bone area/tissue area (BA/TA%) in AD-fed mice (*p* < .0001) with a further statistically significant effect based on cage density (*p* = .0005) driven by a substantial reduction in AD-fed 1/cg compared to AD-fed 5/cg (Figure 6E). Changes in BV/TV% were primarily due to AD-fed specific reduction in cortical thickness (Ct.Th) compared to Cas-fed mice (*p* < .0001; Figure 6F). Due to the observation of porosity in patient bones with CKD, we measured the extent of cortical porosity in our samples. Consistent with CKD, there was a significant diet effect with the 2-way ANOVA (*p* = .0057); yet, there were no significant differences calculated within the different density groups (Figure 6G). With dynamic histomorphometry of the femur midshaft we noted no significant changes in endocortical bone formation rates (Ec.BFR) whereas AD-fed 1/cg group demonstrated a significant reduction in periosteal BFR compared to Cas-fed 1/cg mice (Figure S1B and C). These data demonstrate that 4 wk of CKD and differing cage density ultimately affect bone loss in a compartment specific manner.

**Figure 6:**
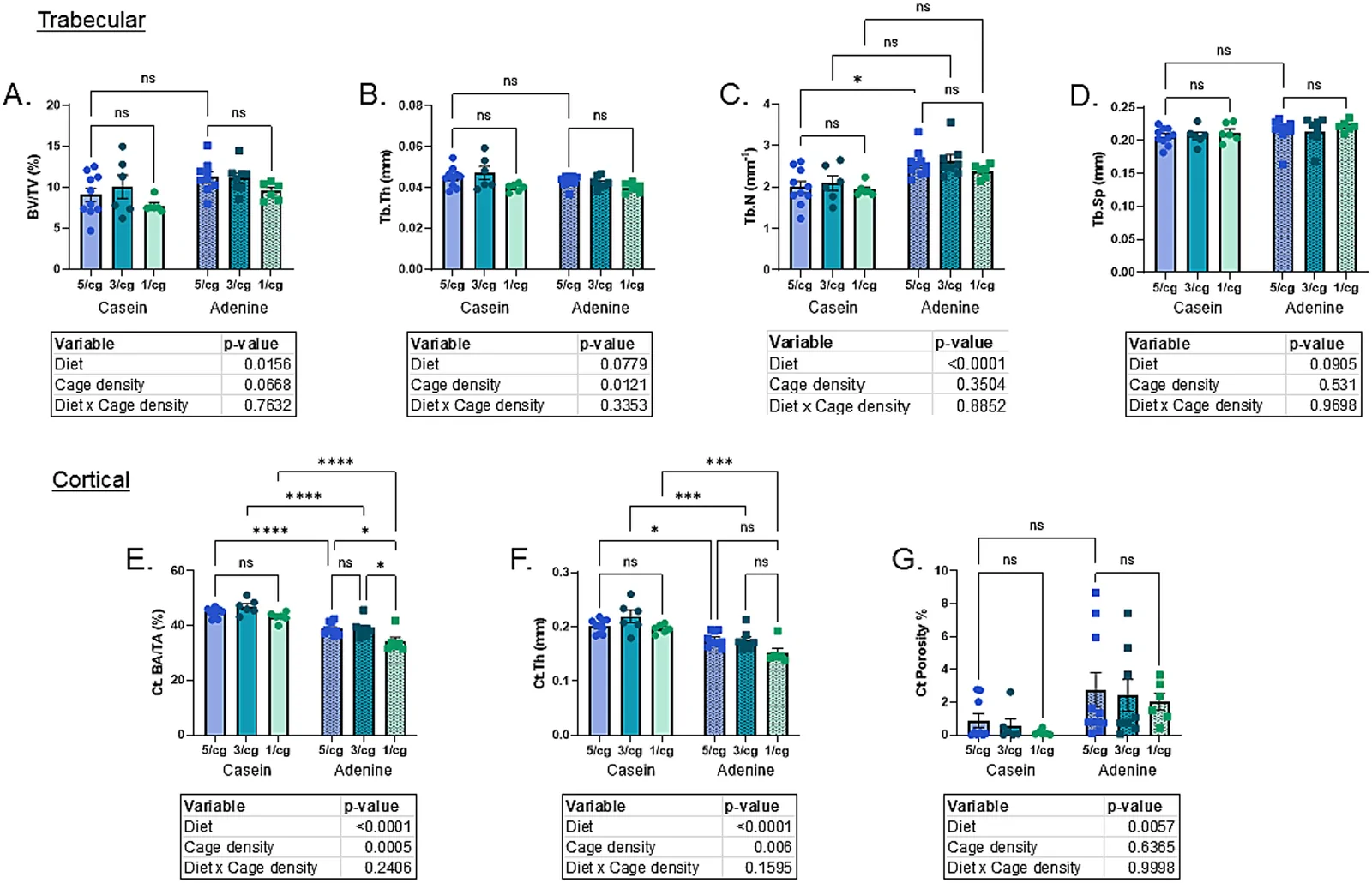
Cage density promotes primarily cortical bone loss with minimal changes in trabecular bone. Micro-CT analysis of the distal femur after 4 wk of diet administration of the trabecular compartment for (A) bone volume vs tissue volume (BV/TV %), (B) trabecular thickness (Tb.Th.), (C) trabecular number (Tb.N.), and (D) trabecular separation (Tb.Sp.). Micro-CT was also used to measure cortical bone of the femur midshaft for (E) cortical bone area vs tissue area (BA/TA %), (F) cortical thickness (Ct.Th.), and (G) cortical porosity. Corresponding 2-way ANOVA tables are listed below (*n* = 5-10 per group; ^*^p < .05, ^**^p < .01, ^***^p < .001, and ^****^p < .0001).

The final arm of CKD-MBD is incidence of cardiovascular calcifications: the aberrant ectopic and detrimental deposition of calcified material within the heart or large vessels. Due to the elevations in serum phosphorus, we analyzed coronal heart sections with von Kossa to highlight calcium deposits along with Masson’s Trichrome to visualize incidence of accumulation of fibrotic material. As expected, hearts from Cas-fed mice remained normal with no observations of fibrotic or calcified material, regardless of cage density (Figure 7). Additionally, hearts evaluated from the AD-fed 3/cg were also visually clear of fibrosis and calcifications (Figure 7). Interestingly, hearts from AD-fed 1/cg mice showed calcification deposits in 5/10 of the total samples, all of which aligned with areas of modest fibrosis (Figure 7). This is combination of two independent AD-fed 1/cg collected cohorts, whereby 2/5 mice in cohort 1 and 3/5 in cohort 2 showed gross indices of cardiac calcification. These results demonstrate cage density has a profound impact on the incidence of cardiac calcification during CKD.

**Figure 7:**
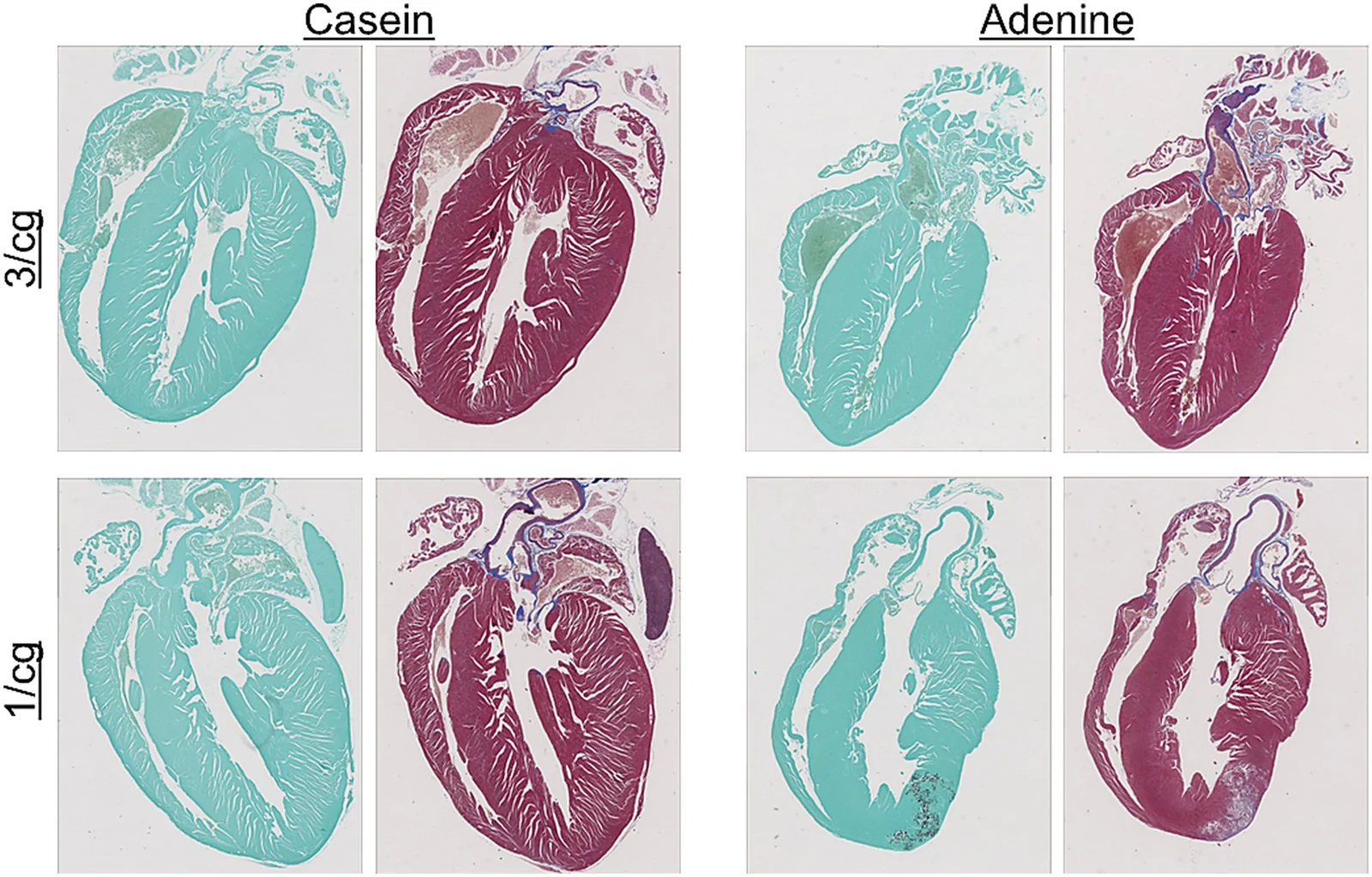
Cage density enhances heart calcifications. Coronal heart sections were stained for von Kossa (left) and Masson’s trichrome (right) to detect calcifications and fibrosis, respectively. Representative images for Cas-fed 3/cg, Cas-fed 1/cg, AD-fed 3/cg, and AD-fed 1/cg are included for each stain. All images are 4× magnification.

## Discussion

Animal models are germane to the progression of understanding physiologically normal processes and pathogenesis of disease for discovering novel therapies, especially as they serve as the critical intermediary between in vitro molecular studies and applications in humans.^19^ Mineral and bone disorder of CKD occurs due to the pathogenic changes in multiple systems; thus, effects of tissue cross-communication cannot be fully replicated in isolated cell culture arrangement.^20^ Mouse models are an attractive and frequently used research tool in this field due to their rapid colony expansion, ease for genetic manipulation, and parallel disease outcomes. It is important to recognize caveats still exist when working with mouse models, and this is the first study to empirically determine cage density exerts a significant influence on progression and outcomes during the AD-induced model of CKD.

Our first observation for effect of cage number was found in the body weight, especially those maintained on the CKD-inducing AD. Casein control fed animals were relatively unaffected by cage density, which is consistent with other studies evaluating housing numbers. Indeed, work by Smolensky et al., demonstrated that when mice are maintained on standard chow, there were no differences in body weight regardless of the number of animals in the cage.^21^ Adenine-diet has been previously shown to cause body weight loss.^5^ In our study, the effect was much more profound and significantly different when adenine-fed mice were individually housed, compared to the same diet-fed animals housed at maximum density of 5 per cage. This aligned with anecdotal evidence from the study by Promruk et al. In their attempt to perform pair feeding, adenine-fed and individually housed animals showed a robust 30% weight loss, and the experiment was discontinued due to humane reasons.^22^ Similarly, body condition score used as a means to evaluate wellbeing of an animal and was reduced in our study within the adenine-fed groups in a density-dependent manner. Parallel to effects observed in patients with CKD, adenine-fed mice exhibited perturbations in serum biochemistry parameters indicative of decreased kidney function. This included elevations of serum BUN and phosphate. Interestingly, there was alignment with weight loss and the further elevated BUN and phosphate in the AD-fed 1/cg group compared to the AD-fed 5/cg mice. Thus, it is possible that these worsened parameters contributed to the overall body phenotype. Importantly, in measuring food intake, we noted that mice housed at 1/cg consumed significantly more food, regardless of the type of diet, compared to those housed at 5/cg fed the same diet for the first 2 wk of the study. By 3 wk, the 1/cg groups were consuming similar amounts of food to the 5/cg mice on the same diet. Importantly, the AD-fed mice maintained lower intake than Cas-fed mice. For adenine-fed mice, these 2 wk of increased food intake early in the study could contribute to the worsened kidney function due to elevated adenine accumulation facilitating more renal damage. At the RNA level, kidney markers for fibrosis and inflammation were similar between all adenine-fed groups. Histological evaluation demonstrated minimal differences between cage densities for positive fibrosis staining area. Interestingly, we did observe significant increases in von Kossa stain, indicative of calcifications, for the AD-fed 1/cg mice compared to the AD-fed 5/cg group. We postulate enhanced mineral accumulation due to the accelerated bone loss is likely responsible for this observation.

Indeed, pronounced bone loss could contribute to elevated serum phosphate due to breakdown and release of hydroxyapatite. Bone quantity is known to be negatively impacted by CKD, which we observed was also further compounded in a cage housing number specific manner. Interestingly, we also noted this was also compartment specific, as trabecular bone was relatively unaffected by cage density. The initial study reporting adenine-mediated CKD in mice showed that after 8 wk of diet administration, the trabecular compartment exhibited increased bone.^5^ Of our micro-CT trabecular measurements, only trabecular number was found significantly different in the AD-fed 5/cg compared to the casein-fed controls. It is possible that the shorter 4-wk diet duration was not sufficient to fully replicate the changes observed at 8 wk. Our bone RNA analysis after 4 wk of diet administration did not illuminate major effects with osteoblast gene expression. Only osteocalcin (*Bglap*) and *Fgf23* mRNA exhibited a diet effect. *Bglap* mRNA was lower and *Fgf23* mRNA was increased in the AD-fed animals, the latter consistent with response to increased serum phosphate circulation in these mice.^23^^,^^24^ However, there were no cage number effects for these genes. Secondary hyperparathyroidism frequently occurs in the later stages of CKD aligning with the results in our study with elevated PTH in the adenine-fed mice.^25^^,^^26^ PTH signaling, especially in a chronic fashion, is known to promote bone loss through enhancing osteoclastogenesis.^27^ However, despite the significant rise in serum PTH levels in the adenine-fed mice, there were no differences between the cage number groups. In our von Kossa-stained bone sections, bone marrow adipocytes could be observed, appearing as large white circles within the marrow space. Patients with CKD have been noted to exhibit increased bone marrow adipose tissue (BMAT), yet the mechanism is unclear.^28^ These adipocytes, unique to this niche, can secrete adipokines which negatively impact bone formation.^29–31^ Bone marrow adipose tissue increases from caloric restriction, cold temperatures, and aging.^32–34^ Promruk et al. found BMAT expansion could not be attributed to calorie intake.^22^ Temperature is an interesting avenue as mice are typically housed in temperatures lower than their thermal neutral (25 °C vs 34 °C, respectively).^35^ Although housing in 34 °C could prevent ovariectomy-induced bone loss,^35^ increased temperature towards thermoneutral was unable to prevent bone loss for male mice housed individually.^36^ The latter is hypothesized to be due to neurological changes resulting from social isolation.^12^ Thus, further work is required to refine the mechanism of bone loss during CKD, especially in these differing housing conditions, whether it be primarily mediated with BMAT-driven inhibition of bone formation, osteoclast bone resorption, or a potential neurological component.

One of the more interesting observations in our study was the incident of cardiac calcifications arising only in the mice provided AD and housed individually. Mice utilized in this study were the C57Bl6J strain, prolifically used as WT mice in a multitude of translational studies as well as background strain for genetically modified animals. Importantly, this strain maintains a functional *Abcc6* gene known to significantly blunt ectopic calcifications.^37–39^ Thus, to facilitate all aspects of CKD-MBD using this background strain, including the cardiovascular calcifications, phosphate content must be manipulated 2-3 times above the physiologically normal level (1.2%-2.0% vs 0.6%).^40–42^ In CKD, serum phosphate and calcium are constantly monitored owing to the propensity for crystal formation at high concentrations. Indeed, dietary and pharmacological strategies have been developed to help reduce phosphate uptake and burden in subjects with CKD.^43–47^ Even with only 4 wk of diet administration, AD-fed 3/cg and AD-fed 1/cg mice showed elevated phosphate over the AD-fed 5/cg yet were not different between the groups, suggesting other factors are necessary for calcification deposition. FGF23 is the critical phosphaturic hormone and increases during CKD in response to the gradual accumulation of circulating phosphate. Although AD-fed mice at 5/cg and 3/cg showed significant increases in serum iFGF23 over casein, AD-fed 1/cg mice did not exhibit the same magnitude of elevated serum iFGF23. Previous studies found FGF23 to be protective during CKD as genetic loss or inhibition of FGF23 led to increased cardiovascular calcifications^48^ and increased mortality.^49^ Thus, it is possible impaired phosphate excretion in the AD-fed 1/cg group due to the lack of iFGF23 production contributed to calcifications. These observations, through further investigation, could have important clinical implications, as recent studies demonstrate enhanced cardiovascular complications in socially isolated patients with CKD.^42^^,^^50–52^

The current study design contains several limitations. The first is that only males were used and thus begs the question whether similar effects would be seen in females. Our experimental strategy was based on simulating colony management of a non-commercially generated strain. In this situation, weaning a male mouse to its own cage would preclude any future housing with other males, requiring this mouse to remain individually housed for the duration of a CKD study. Females, in contrast, are rarely noted to have a similar territorial instinct and can be relocated to cages with other non-related females as needed. Thus, the incidence of individually housed females is much lower. Additionally, in the study by Mountain et al., individual housing when fed standard chow had minimal effects on female bones in contrast to males.^12^ The second is that we did not test any other model of CKD. Multiple models exist that establish CKD through surgical techniques, genetic modifications or exogenous interventions with diets or pharmacologic agents.^4^ The growing utilization of the AD and our previous experience with this approach is why we pursued analysis with this methodology. As our data implies, due to differential outcomes in male mice with CKD based on cage density housing, it would be prudent to consider that variable when establishing experimental designs for any other CKD model. The final limitation was the short duration of the study. Although we observed cage density trends in various parameters, there were minimal significant differences observed between the AD-fed 3/cg and 5/cg mice, likely due to the short 4-wk duration. This time point was chosen to ensure direct comparison between all the groups within the experiment. Survival curves were not a primary outcome for the study and any further reduction of body score in the AD-fed 1/cg mice would have initiated humane euthanasia requests within the animal facility. Additional experiments using extended timelines would be needed to determine if AD-fed 3/cg further separated from AD-fed 5/cg in their CKD progression and mineral homeostasis.

In summary, we found individually housing males facilitated progression of CKD. This was reflected with exacerbated body weight loss and reduced body condition score. Individually housed mice with CKD also exhibited highest concentrations in BUN and exclusively incurred cardiovascular calcification. This is a critical variable for basic and translational CKD murine studies.

## Supplementary Material

26_-_09_-_14_Supplementary_Figure_S1_ziag160

## Data Availability

All reasonable requests to review the datasets presented herein can be made through contacting the corresponding author after official publication.
