# Supplementary material for: Negative impact of single housing on chronic kidney disease mineral and bone disorder outcomes
Source: JBMR Plus. 2026 Oct 24;10(11):ziag160. doi: 10.1093/jbmrpl/ziag160 (PMC13695957; doi:10.1093/jbmrpl/ziag160)
Supplement: Supplementary file 1 [file 26_-_09_-_14_supplementary_figure_s1_ziag160.docx]

**Supplementary Figure S1.** **A**. Representative distal femur images stained with von Kossa. All images are 4X magnification. Dynamic histomorphometry quantification of **B**. periosteal bone formation rate per bone surface (Ps.BFR/BS) and **C.** endocortical bone formation rate (Ec.BFR/BS). (n=3-6; *p<0.05, **p<0.01)
